# Supplementary material for: Two-variable nullcline analysis of ionic general equilibrium predicts calcium homeostasis in ventricular myocytes
Source: PLoS Comput Biol. 2020 Jun 5;16(6):e1007572. doi: 10.1371/journal.pcbi.1007572 (PMC7316341; doi:10.1371/journal.pcbi.1007572)
Supplement: S1 File — In this supplemental information file, we give a full description of the rabbit model, develop the nullcline analysis, mentioned in the results section, using total calcium concentrations in the cell instead of free calcium concentrations. We also provide additional details on how SERCA uptake changes affect cell behavior concerning the physiological mechanism for SERCA gene therapy failure, as indicated in the discussion section. This supplemental file also includes an appendix with tables of the different parameters used in the models. (PDF) [file pcbi.1007572.s001.pdf]

Supplemental Material

D. Conesa[1], B. Echebarria[1], A. Peñaranda[1], I.R. Cantalapiedra[1], Y. Shiferaw[2], and E. Alvarez-Lacalle[1]

[1] *Departament de Física. Universitat Politècnica de Catalunya-BarcelonaTech. Barcelona. Spain.*
[2] *Physics Department. California State University Northridge. Los Angeles. United States.*

Contents

In this supplemental material, we provide supplemental information about four features:

- Full description of the rabbit ventricular model.
- We show the analysis described in the text using total calcium in the cell together with calcium in the SR.
- We provide additional details on how SERCA uptake changes affect cell behavior in relation to the physiological mechanism for SERCA gene therapy failure.
- We provide an appendix with tables of the parameters used in the model.

Rabbit ventricular model

General structure and voltage clamp

As described in the main manuscript we consider the cell to be a 3-dimensional array composed of Calcium Release Units (CaRUs) with cuboid form ($dx \times dy \times dz$ where $dx = dy \neq dz$), each one divided into volumes associated with RyR cluster. The 5 compartments or subvolumes of each CaRU are cytosol (i), subsarcolemma (s), network sarcoplasmic reticulum (nsr), junctional sarcoplasmic reticulum (jsr) and dyadic junction (d). This model considers the diffusion of calcium ions between compartments of the same CaRU, as well as diffusion between neighboring CaRUs. Moreover, calcium buffers are taken into account, thus differentiating between free and bound to buffers calcium ions. A full description of the dynamics of calcium in the CaRUs has already been explained in the main manuscript but is repeated here for completeness.

$$\frac{dc_d}{dt} = j_{RyR} - j_{LCC} - j_{ds} \tag{1}$$
$$\frac{dc_s}{dt} = j_{NCX} - j_{si} + \frac{v_d}{v_s} j_{ds} + D_s \nabla^2 c_s \tag{2}$$
$$\frac{dc_i}{dt} = -j_{SrCa} + \frac{v_s}{v_i} j_{si} - j_{buff_i} + D_i \nabla^2 c_i \tag{3}$$
$$\frac{dc_{jsr_{TOT}}}{dt} = j_{tr} - \frac{v_d}{v_{jsr}} j_{RyR} \tag{4}$$
$$\frac{dc_{nsr}}{dt} = -j_{tr} + \frac{v_i}{v_{nsr}} j_{SrCa} + D_{sr} \nabla^2 c_{nsr} \tag{5}$$

The main currents are due to L-type calcium channels which introduce calcium in the dyadic space, j_{LCC}; the flux across RyR which also goes into the dyadic from the junctional SR, j_{RyR}; the flux due to SERCA pumps, j_{SrCa}, from the cytosol to the SR and the Na^+/Ca^{2+} exchanger which leads to fluxes from the subsarcolemma to the exterior of the cell, j_{NCX}. Currents related with the LCC and the Na^+/Ca^{2+} exchanger are voltage dependent. We pace externally the cell using a clamped action potential with the form

$$V(t) = \begin{cases} (V_{max} - V_{rest})\sqrt{1 - (\frac{\widetilde{t}}{APD})^2} + V_{rest}, & \text{if } \widetilde{t} < \text{APD} \\ V_{rest}, & \text{if } \widetilde{t} > \text{APD} \end{cases} \tag{6}$$

where $\widetilde{t} = t - nT$, with n = 0, 1, 2... Voltage affects the calcium intake via LCC, j_{LCC}, since increases in voltage open the channel while extrusion of calcium via j_{NCX} is favored when voltage is down. The details of this voltage-dependence will be described in subsequent sections.

Regarding diffusion, we take the diffusion constant in the cytosol, D_i, to be the same as in the subsarcolemma, D_s, and ten times larger than in the SR, D_{SR}, as can be seen in the table of the appendix B. Given that the CaRU is not a cube, the in-plane direction and the across Z-plane direction have different time constants related with diffusion. The characteristic distances between units in the Z-plane plane are 500 nm. Across planes we take a characteristic distance of dz_{ef} = 1800 nm, a slightly lower effective distance that dz = 2000 nm given the presence of t-tubules.

Calcium extrusion (Na^+/Ca^{2+} Exchanger) and intake (LCC)

The current associated to Na^+/Ca^{2+} exchanger depends on intracellular and extracellular Na^+ concentrations ($[Na]_i$ and $[Na]_o$ respectively), which are set as constant in the voltage-clamped model, as well as on extracellular Ca^{2+} concentration ($[Ca]_o$), also constant, and subsarcolemmal calcium concentration c_s. Its expression reads

$$j_{NCX} = \frac{g_{NaCa}}{1 + (K_{da}/c_s)^3} \frac{e^{\eta z}[Na]_i^3[Ca]_o - e^{(\eta-1)z}[Na]_o^3 c_s}{S(c_s)(1 + k_{sat} e^{(\eta-1)z})} \tag{7}$$

where the $S(c_s)$ function is

$$\begin{aligned} S(c_s) =& [Na]_o^3 c_s + K_{mNao}^3 c_s [1 + (c_s/K_{mCai}] + K_{mCao}[Na]_i^3 + \\ &+ [Na]_i^3[Ca]_o + K_{mCai}[Na]_o^3[1 + ([Na]_i/K_{mNai})^3] \end{aligned} \tag{8}$$

and z=$\frac{VF}{RTemp}$, with V being the membrane voltage, F the Faraday constant, R the ideal gas constant and $Temp$ the temperature. The different parameters are defined in the appendix.
The current produced by the LCC channels taking calcium from the extracellular medium towards the intracellular depends on the voltage, the extracellular Ca^{2+} concentration and the calcium in the dyadic volume, the one close to both the RyR cluster and the LCC channels. The current is proportional to the number of LCC channels in open state (O_{LCC}). We use 5 possible states for each channel: O for the open state, C1 and C2 for closed states, and I1 and I2 for inactivated states. In each CaRU we consider a group of 5 LCC channels. We track the state of each one of the channels using the length rule to establish a transition between states with the uniformly distributed random number generator RANMAR. The expression of this current reads

$$j_{LCC} = g_{CaL} O_{LCC} 4 z_m \frac{e^{2z} c_d - [Ca]_o}{e^{2z} - 1} \tag{9}$$

where z is still $z = \frac{VF}{RTemp}$ and $z_m = 0.341zF$. The transition rates depend on voltage and Ca^{2+} concentrations. These transition rates are named as indicated in the main manuscript:

$$a_{12} = p_\infty \tag{10}$$
$$a_{21} = 1 - p_\infty \tag{11}$$
$$a_{45} = (1 - p_{if})/3 \tag{12}$$
$$a_{24} = 0.00413 + 0.05 f_{ca} \tag{13}$$
$$a_{34} = 0.00195 + 0.025 f_{ca} \tag{14}$$
$$a_{15} = p_o/\tau_o + (p_o/\tau_{ca}) f_{ca} \tag{15}$$
$$a_{51} = (1 - p_o)/\tau_o + (1 - p_o)/\tau_{ca} \tag{16}$$
$$a_{43} = a_{34}(a_{23}/a_{32})(a_{42}/a_{24}) \tag{17}$$
$$a_{54} = a_{45}(a_{51}/a_{15})(a_{24}/a_{42})(a_{12}/a_{21}) \tag{18}$$
$$p_\infty = \frac{1}{1 + e^{-V/8}} \tag{19}$$
$$\tau_o = (\rho_o - 450) p_{of} + 450, \quad \rho_o = 10 + 5000 e^{V/15.6}, \quad p_{of} = 1 - \frac{1}{1 + e^{-(V+20)/4}} \tag{20}$$
$$p_{if} = \frac{1}{1 + e^{-(V+20)/3}} \tag{21}$$
$$f_{ca} = \frac{1}{1 + (K_{LCC}/c_d)^3} \tag{22}$$
$$T_{ca} = \frac{78 + 0.1(1 + (c_d/K_{LCC})^4)}{1 + (c_d/K_{LCC})^4)}, \qquad \tau_{ca} = (\rho_o - T_{ca}) p_{of} + T_{ca} \tag{23}$$
$$p_o = \frac{1}{1 + e^{-(V+20)/11.32}} \tag{24}$$

a_{23}, a_{32} *and* a_{42} are set as constants. Their values are defined in table of parameters in Appendix.

Calcium release (RyR) and uptake (SERCA)

The calcium release current, through RyR, is like a diffusive current, but depending not only on the difference among c_{jsr} and c_d but also on the conductivity of RyR and, a non-deterministic feature of the model, the number of these proteins in the open state (O_{RyR}).

$$j_{RyR} = g_{rel} O_{RyR} (c_{jsr} - c_d) \tag{25}$$

In each CaRU we consider a cluster of $N_{RyR} = 40$ RyR, where each one of the receptors can be in one of the 4 possible states: O for the open state, C is the closed state, while I1 and I2 are two inactivated/terminated states.

Their dynamics are treated stochastically as a function of transition rates k_{ss^*}, with s the previous state and s^* the state after the transition. At each time step, from the current configuration of states, the next configuration is randomly chosen following a Markov chain process using the RANMAR algorithm as uniformly random number generator used to obtain binomial numbers to decide how many RyR in the state s change to the different states s^*.

Regarding the transition rates, $k_{co} = k_{i2i1}$, and $k_{ci} = k_{oi}$ depend on calcium concentrations in junctional sarcoplasmic reticulum and dyadic junction at each time step with different cooperativities, while the rest of the rates are set as constants following the equalities $k_{ic} = k_{io}$ and $k_{oc} = k_{i1i2}$.

$$k_{co} = k_p \frac{2K_{jsr}^2 + c_{jsr}^2}{K_{jsr}^2 + c_{jsr}^2} \frac{c_d^2}{K_{oc}^2 + c_d^2} \tag{26}$$

$$k_{ci} = k_i \frac{2K_{jsr}^2 + c_{jsr}^2}{K_{jsr}^2 + c_{jsr}^2} \frac{c_d}{K_{in} + c_d} \tag{27}$$

where $K_{in} = 50\mu M$, $K_{oc} = 20\mu M$ and $K_{jsr} = 450\mu M$.

For completeness, we also recall here that the current given by the SERCA pump is thermodynamically limited. It is considered to depend only on Ca^{2+} concentrations in cytosol and network SR.

$$j_{SrCa} = \nu_{up} \frac{(c_i/K_i)^2 - (c_{nsr}/K_{sr})^2}{1 + (c_i/K_i)^2 + (c_{nsr}/K_{sr})^2} \tag{28}$$

where K_i and K_{sr} are half occupation binding constants, and ν_{up} the maximum uptake strength.

Calcium buffer formulation

Buffers are located by construction in the cytosol and in the junctional SR where calcium can bind to them. As explained in the main manuscript, buffers in the cytosol and in the SR have a different formulation. In the SR, there is only calsequestrin (CSQ) as a buffer and only close to the RyR in the jSR volume. We compute the evolution of $c_{jsr_{TOT}}$, which includes both free calcium in jSR (c_{jsr}) and calcium bound to CSQ. Once the total amount of calcium is computed in the jSR, this calcium is split between the free and buffered parts using the fast-buffering approximation.

$$c_{jsr_{TOT}} = c_{jsr} + \frac{c_{jsr} B_{CSQ}}{c_{jsr} + K_{CSQ}} \tag{29}$$

where B_{CSQ} is the concentration of calsequestrin in the jsr and K_{CSQ} its dissociation constant. The free calcium concentration c_{jsr} can be obtained solving the quadratic equation

$$c_{jsr} = \frac{1}{2}(c_{jsr_{TOT}} - (K_{CSQ} + B_{CSQ}) + \sqrt{(K_{CSQ} + B_{CSQ} - c_{jsr_{TOT}})^2 + 4K_{CSQ}c_{jsr_{TOT}}}) \quad (30)$$

In the cytosol there are buffering currents in the model which depend on binding (k_{on}) and unbinding (k_{off}) rates of calcium in the buffers b, and also on the concentration of buffers, B_b, and concentration of Ca^{2+} bound to them, c_b. These currents are computed by adding the contribution of all the buffers in the compartment

$$j_{buff_i} = \sum_b j_b = \sum_b k_{on_b} c_i (B_b - c_b) - k_{off_b} c_b \quad (31)$$

while Ca^{2+} concentration bound to buffer b, c_b follows the equation

$$\frac{dc_b}{dt} = k_{on_b} c_i (B_b - c_b) - k_{off_b} c_b = j_b \quad (32)$$

where j_b is the contribution of buffer b to the buffering current j_{buff_i}. Notice that we do not use the rapid buffering approximation in any of the buffers of the cytosol. The reason is that fast buffering approximation leads to a loss of mass in any type of propagation algorithm we have considered. The lack of conservation of mass in models with fast buffering approximation seems to be not known in the literature but it is clear that any Euler algorithm does not conserve mass to all orders. Using fluxes we can enforce with the Euler's algorithm that the exact amount of calcium that leaves one compartment place is placed in another compartment, in a buffer or goes outside of the cell. All in all, the changes in total calcium in the cell come exactly from the differences between the calcium that enters the cell and the amount that leaves it.

We consider five buffers in the cytosol: the buffering which is proper of the SERCA pump, Calmodulin (CAM), buffers in the internal membrane structures (SLH), Tropomyosin C (TnC) and the dye buffering which we take to be similar in properties to Fluo-4. We do not take into account any buffer in the dyadic volume since they are considered to be non-existent there and we neglect the effect of buffers in the subsarcolemma for simplification. The parameters are given in the appendix table.

General equilibrium using total cell calcium concentration as a variable

Averaged variables and reduction in dimensionality

The main manuscript uses averaged values of currents but also, in the reduction of the dimensionality, it states that averaged internal variables which are not calcium concentrations have very fast dynamics. We define here these averaged quantities. They are related to the state of RyR and LCC channels, and Ca^{2+} concentrations attached to cytosolic buffers. Since all CaRUs behave, on average, in the same way in our ventricle cell model, when we set the initial condition for the cytosol and SR to obtain the surfaces (which compute the calcium in and out of the cell and in and out of the SR), we set these averaged quantities to their equilibrium value, the same for all CaRUs. The averaged quantities we define are: L_{LCC} as the fraction of occupation in each one of the 5 possible states for LCC (L = C1, C2, O, I1, I2), R_{RyR} as the fraction of occupation for the 4 states of RyR (R = C, O, I1, I2), and c_b as the calcium attached to each

one of the buffers we take into consideration. With n being an index to label a CaRU and N the total number of CaRUs, they read

$$L_{LCC} = \frac{1}{N}\sum_{n=1}^{N} L_{LCC}^{n} \tag{33}$$

$$R_{RyR} = \frac{1}{N}\sum_{n=1}^{N} R_{RyR}^{n} \tag{34}$$

$$c_b = \frac{1}{N}\sum_{n=1}^{N} c_b^{n} \tag{35}$$

We take all initial states to be the same in the different CaRUs and we check that after one beat these averaged quantities are indeed in local equilibrium. This sustain the claim that a huge reduction in dimensionality is possible.

We can define now the average free concentration of calcium in compartment x as:

$$c_x = \frac{1}{N}\sum_{n=1}^{N} c_x^{n} \tag{36}$$

$$\tag{37}$$

From the averaged concentrations, we can define the calcium concentration per volume of cytosol in 2 differentiated regions: the basal region, which includes cytosol, subsarcolemma and dyadic junction, whose calcium concentration will be Q_I, and the sarcoplasmic reticulum region, comprising network and junctional SR, with concentration Q_{SR}. The total calcium concentration is Q_{TOT}, the sum of the 2 regions:

$$Q_I = \frac{N}{v_i}[v_i(c_i + c_{buff_i}) + v_s c_s + v_d c_d] \tag{38}$$

$$Q_{SR} = \frac{N}{v_i}[v_{nsr}c_{nsr} + v_{jsr}(c_{jsr} + c_{CSQ})] \tag{39}$$

$$Q_{TOT} = Q_I + Q_{SR} \tag{40}$$

From this, we can define the total Ca^{2+} concentration per volume of cytosol in a CaRU, in average, as

$$c_{TOT} = Q_{TOT}/N \tag{41}$$

We define a similar Ca^{2+} concentration per volume of cytosol for free calcium ions in the SR

$$c_{sr} = \frac{1}{v_i}(v_{nsr}c_{nsr} + v_{jsr}c_{jsr}) \tag{42}$$

The claim we make in the paper is that that the extrusion and intake of calcium in the cell during one beat is basically dependent on these averaged quantities. More specifically, given equilibration in the cytosol and in the SR, knowing two of those averages at the pre-systolic time will be sufficient to determine the extrusion/intake of calcium during the whole beat. Similarly, after one period, the final state does not depend on the initial state of any other variable, given

that they are fast compared with the pacing period, and therefore they can be neglected in our analysis. We show this now explicitly in Figure A, where we compute the end-diastolic value of SR calcium concentration after a period, starting from different initial values of the initial calcium buffer occupancy of TnC and CAM, and in the fraction of initial open RyRs. Figure A shows that the final state is completely independent of the initial state of the buffers, and depends very slightly on the initial state of the RyRs, as long as it is close to its value at the equilibrium point which is generally recovered at the beginning of the external stimulation. The reason for this slight dependence on the state of the RyR, contrary to what happens with the buffers, is that the release depends very sensitively on the number of ready to open states of the RyR. The dynamics of both the RyR and LCC channels is fast (100ms for the RyR) compared with the pacing period, but they do not have time to equilibrate in the time between the external excitation and the initiation of release (20ms). Thus, starting with values very far from the equilibrium value would affect the equilibrium surfaces. In any case, the fact that the predictions of the maps (or general equilibrium model) agree very well with the numerical results obtained integrating the dynamical equations is an indication that the dimensionality reduction works.

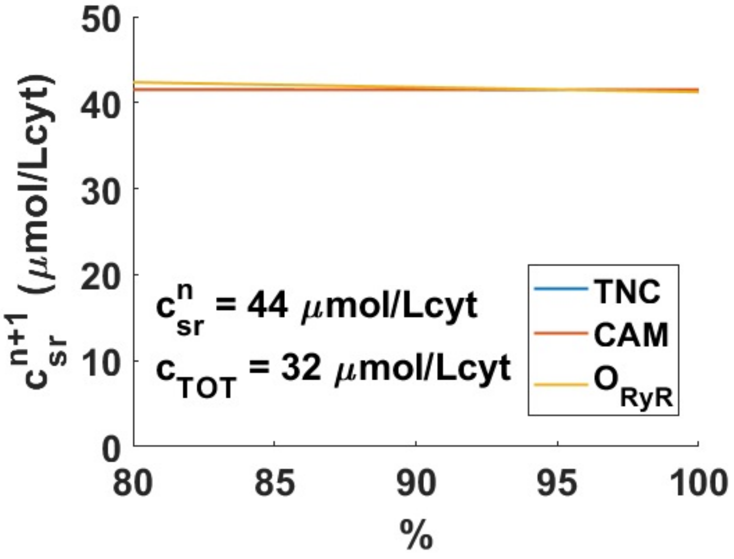

Figure A: Value of SR calcium concentration after one stimulation, starting from the same initial value of c^n_{sr}=44 μmol/Lcyt, and different initial values of buffer concentration and fraction of open RyRs. The final state is independent on the specific value at the beginning of the stimulation.

Nullclines and homeostasis

The key objects to understand homeostasis in our framework are the nullclines. We take c_{sr} and c_i as the two most relevant variables to determine the 4 key currents that exchange Ca^{2+} into and out of the cell and into and out of the SR. In terms of the nomenclature of the previous section we have:

$$\Delta Q_{in} - \Delta Q_{out} = 0 \qquad (f - nullcline,\ \Delta Q_{TOT} = 0) \qquad (43)$$

$$\Delta Q_{up} - \Delta Q_{rel} = 0 \qquad (g - nullcline,\ \Delta Q_{SR} = 0) \qquad (44)$$

Since we work with f-nullcline (where $\Delta Q_{TOT} = 0$) and g-nullcline (where $\Delta Q_{SR} = 0$), one could think that $c_{_{TOT}}$ and c_{sr} are more directly related with the nullclines than the free cytosolic concentration. However, in the main manuscript we use the variable c_i and c_{sr} instead to describe the state of the system. We must clarify here that both are completely equivalent. One can use c_i and c_{sr} to describe the nullclines or $c_{_{TOT}}$ and c_{sr}. The reason is that $c_{_{TOT}}$ is basically a function of both c_{sr} and c_i given that we find that the calcium attached to buffers is very close to equilibrium and that the concentrations in the dyadic space and subsarcolemma space is very similar to the one in the cytosol at the end of diastole ($c_d = c_s = c_i$). Similarly one can write that $c_{jsr} = f(c_{sr})$ and $c_{nsr} = f(c_{sr})$ given that c_{nsr} and c_{jsr} are close to each other at the end of diastole and c_{jsr} is in equilibrium with calsequestrin. In Figure B we show the f- and g- nullclines for our rabbit ventricular cell as a function of $c_{_{TOT}}$ and c_{sr} pre-systolic variables.

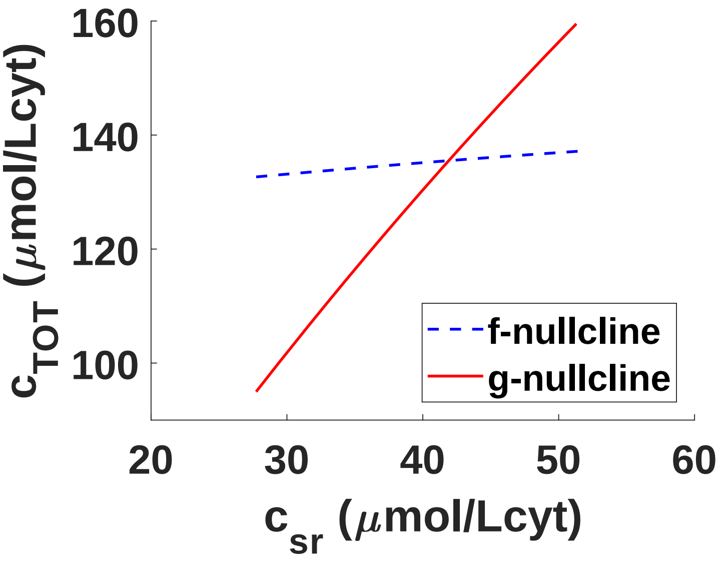

Figure B: f- and g- nullclines as a function of pre-systolic (c_{sr}, $c_{_{TOT}}$) in our rabbit ventricular cell. The f-nullcline presents a slight slope when considered as a function of $c_{_{TOT}}$ and c_{sr}, implying that Q_{TOT} is conserved only within a narrow value of its pre-systolic condition.

It can be observed that f-nullcline shows a slighter slope when considering $c_{_{TOT}}$ instead of c_i as the natural variable. Understanding and analyzing nullcline changes under single shocks seems easier with these variables. The reason is that comparing the g-nullcline and their shocks with a constant f-nullcline rather than with a curve with a more pronounced slope is more straightforward. One can immediately obtain information on whether the shock in one of the nullclines is going to affect strongly the total amount of calcium in the cell. However, in the main manuscript we take c_{sr} and c_i as the two direct variables since they are more commonly used in literature and easier to control in experiments.

Effects of changes in SERCA uptake

Now, we provide further details on the analysis of increasing maximum SERCA uptake. As it can be seen in the main manuscript, in normal rabbit ventricular cell the level of c_i slightly decreases

while the SR increases. The peak of the calcium transient remains roughly constant making the transient effectively larger as can be seen in Fig.C. More relevant to us here is to discuss how the change in SERCA is a double shock which affects both nullclines. In Fig.D upper graphs we show the nullclines for different ν_{up} (0.3, 0.5 and 0.7) using both the (c_i, c_{sr}) description of the nullclines and the (c_{sr},c_{TOT}) description. We clearly see that the total calcium levels in the cell increase thanks to the increase in uptake in the normal rabbit. When increasing ν_{up}, the increase of the SR load is the main responsible for the observed increase of the total calcium, c_{TOT}. More important is to observe that the calcium in the cytosol actually decreases. Here, the SERCA pump acts as expected. It increases the level of calcium in the SR diminishing it in the cytosol while increasing the total amount of calcium in the cell. This allows the system to have a much larger transient as we increase the SERCA uptake.

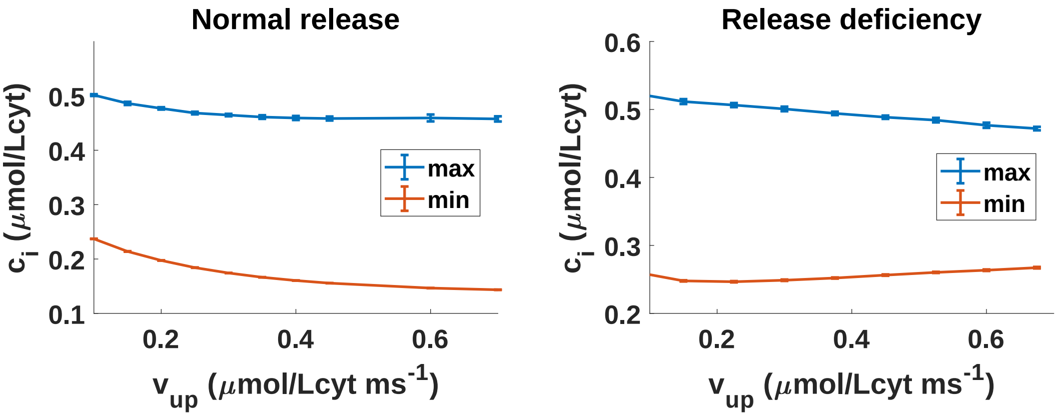

Figure C: Maximum (peak) and minimum Ca^{2+} concentration in cytosol as a function of maximum SERCA uptake ν_{up}, for the normal rabbit (left) and the rabbit cell with release deficiency (right). When increasing maximum uptake, the peak of the transient tends to remain constant for the normal release, while the minimum value slightly decreases. With release deficiency, peak value decreases while minimum increases, implying a lower effective transient.

However, the picture is very different when we consider a cell where the release per unit of RyR is divided by four, as it would be in a deficient release(Fig. D lower). In this case, there is redistribution to the SR calcium (c_{sr}) from the cytosol (c_i) in the basal. In other words, and as stated in the main manuscript, the nullclines show a decrease of c_i when increasing SERCA uptake in the normal release situation, while the nullclines crossing point with release deficiency is located at higher c_i for larger ν_{up}. This difference is observed due to the fact that the nullclines experience different shocks in both cases. The displacement of g-nullcline (balance of calcium in and out of the SR, $\Delta Q_{SR} = 0$) in the case with release deficiency is lower than in the normal situation. Thus, the shock experienced by the f-nullcline (balance of calcium in and out of the cell, $\Delta Q_{TOT} = 0$) makes the homeostatic point to be more loaded in the cytosol. Although increasing total load in both systems, the different behavior in the cytosolic calcium concentration with respect to the one in SR can lead to different beat strength behaviors as a function of SERCA uptake. As explained, this feature can be a key point to understand why SERCA gene therapy may fail.

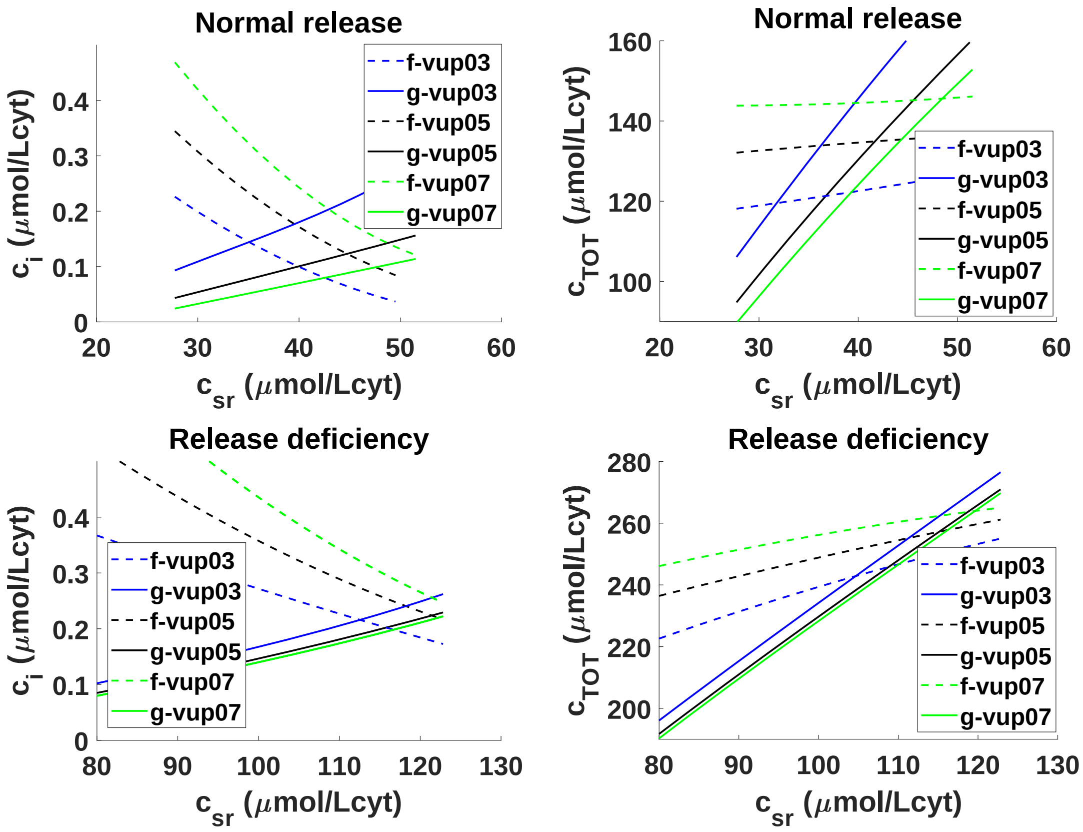

Figure D: f- and g- nullclines for the rabbit ventricular model (above) and the modification with release deficiency (below) for three different values of the maximum SERCA uptake ν_{up}: 0.3, 0.5 and 0.7 $\mu M m s^{-1}$, as a function of (c_{sr}, c_{TOT}) (left) and (c_{sr}, c_{TOT}) (right). Homeostatic c_{sr} is placed at a larger value when increasing the maximum uptake in both situations. Homeostatic cytosolic calcium diminishes when increasing ν_{up} in normal release (upper left), but decreases in release deficiency situation (lower left), while total calcium c_{TOT} shows an increase in both cases, thus meaning a different behavior in the redistribution of calcium inside the cell. Shocks in g-nullcline are less notable with release deficiency, thus making changes to g-nullcline to be the most relevant for the homeostasis.

A Table of Parameters

General parameters		
CaRU size	$dx \times dy \times dz$ (μm^3)	0.5×0.5×2.0
Cell size	$Nx \times Ny \times Nz$	$25 \times 25 \times 50$
Time step	Δt (ms)	0.01
Resting potential	V_{rest} (mV)	-85
Maximum potential	V_{max} (mV)	20
Pacing period	T (ms)	500
Action potential duration	APD (ms)	150
Volume of cytosol	v_i (μm^3)	0.25
Cytosolic volume/nSR volume	v_i/v_{nsr}	25
jSR volume/nSR volume	v_{jsr}/v_{nsr}	1
Volume of subsarcolemma	v_s (μm^3)	0.0245
Volume of dyadic junction	v_d (μm^3)	1.125e-3
Time scale diffusion between dyadic and subsarcolemma	τ_p (ms)	0.02
Time scale diffusion between subsarcolemma and cytosol	τ_s (ms)	0.3
Time scale diffusion between SR and jSR	τ_{tr} (ms)	20
Temperature	Temp (K)	308
Faraday constant	F (C/mol)	96500
Ideal gas constant	R ($JK^{-1}mol^{-1}$)	8.314
Diffusion coefficient in cytosol and subsarcolemma	D_i=D_s ($\mu m^2/ms$)	0.3
Diffusion coefficient in SR	D_{sr} ($\mu m^2/ms$)	0.03
Diffusion rate between cytosolic and subsarcolemma compartments in the Z-plane	D_i/dx^2 (ms^{-1})	1.2
Diffusion rate between cytosolic compartments along z direction	D_i/dz_{ef}^2 (ms^{-1})	0.093
Diffusion rate between subsarcolemma compartments along z direction	Not in contact	0
Diffusion rate between SR compartments in the Z-plane	D_{sr}/dx^2 (ms^{-1})	0.12
Diffusion rate between SR compartments along z direction	D_{sr}/dz_{ef}^2 (ms^{-1})	0.0093
Buffer parameters		
TnC binding rate	k_{onT} (μM^{-1} ms^{-1})	0.0327
TnC unbinding rate	k_{offT} (ms^{-1})	0.0196
TnC concentration in cytosol	B_T (μM)	70
SR-bound buffer binding rate	k_{onSR} (μM^{-1} ms^{-1})	0.1
SR-bound buffer unbinding rate	k_{offSR} (ms^{-1})	0.06
SR-bound buffer concentration in cytosol	B_{SR} (μM)	20
Calmodulin binding rate	k_{onCAM}($\mu M^{-1}ms^{-1}$)	0.035
Calmodulin unbinding rate	k_{offCAM} (ms^{-1})	0.2
Calmodulin concentration in cytosol	B_{CAM} (μM)	24
Fluo-4 binding rate	k_{onFL} (μM^{-1} ms^{-1})	0.07
Fluo-4 unbinding rate	k_{offFL} (ms^{-1})	0.07
Fluo-4 concentration in cytosol	B_{FL} (μM)	50
SLH binding rate	k_{onSLH} (μM^{-1} ms^{-1})	0.1
SLH unbinding rate	k_{offSLH} (ms^{-1})	0.03

SLH concentration in cytosol	B_{SLH} (μM)	15
Calsequestrin concentration in jSR	B_{CSQ} (μM)	3500
Calsequestrin unbinding/binding rate constant	K_{CSQ} (μM)	650
Exchanger parameters		
Uptake strength of exchanger	g_{NaCa} (μM ms^{-1})	10
Extracellular calcium concentration	$[Ca]_o$ (mM)	1.8
Extracellular sodium concentration	$[Na]_o$ (mM)	136
Intracellular sodium concentration	$[Na]_i$ (mM)	9
Saturation constant	k_{sat}	0.27
Voltage sensitivity constant	η	0.35
Inactivation constant	K_{da} (μM)	0.2
External sensitivity constant for Na	K_{mNao} (mM)	87.5
External sensitivity constant for Ca	K_{mCao} (mM)	1.3
Internal sensitivity constant for Na	K_{mNai} (mM)	12.3
Internal sensitivity constant for Ca	K_{mCai} (mM)	0.0036
SERCA parameters		
Maximum uptake of SERCA	ν_{up} (μM ms^{-1})	0.5
Half occupation of cytosolic calcium binding states	K_i (μM)	0.5
Half occupation of SR calcium binding states	K_{sr} (mM)	2.6
LCC parameters		
Strength of the effective flux rate of a single channel	g_{CaL}(μM C^{-1}ms^{-1})	2.508
Number of LCC per CaRU	N_{LCC}	5
Transition rate for opening from C1	a_{23} (ms^{-1})	0.3
Transition rate for closing to C1	a_{32} (ms^{-1})	2
Transition rate for recovery from inactivation I1 to C1	a_{42} (ms^{-1})	0.00224
Threshold for Ca-induced transition	K_{LCC} (μM)	3
RyR parameters		
Number of RyR per CaRU	N_{RyR}	40
Recovery rate from inactivation to close	k_{ic} (ms^{-1})	0.01
Inactivation rate parameter	k_i (ms^{-1})	0.25
Closing rate	k_{oc} (ms^{-1})	0.1
Opening rate parameter	k_p (ms^{-1})	0.4
Single channel strength	g_{rel} (ms^{-1})	0.4
Single channel strength with release deficiency	g_{rel} (ms^{-1})	0.1

Table A: System parameters for our rabbit ventricular model.

A.1 Modified Rabbit Model

Here we expose the differences, in SERCA and buffers, between the normal rabbit ventricular model and the modified rabbit model used when changing g_{CaL}, in Fig.1 in the main manuscript.

Buffer parameters		
Fluo-4 binding rate	$k_{on_{FL}}$ (μM^{-1} ms^{-1})	0.04
Fluo-4 unbinding rate	$k_{off_{FL}}$ (ms^{-1})	0.04
Fluo-4 concentration in cytosol	B_{FL} (μM)	2
SLH concentration in cytosol	B_{SLH} (μM)	0

Calsequestrin concentration in jSR	B_{CSQ} (μM)	3000
SERCA parameters		
Maximum uptake of SERCA	ν_{up} (μM ms^{-1})	0.3
Half occupation of cytosolic calcium binding states	K_i (μM)	0.2
Half occupation of SR calcium binding states	K_{SR} (mM)	2.5

Table B: System parameters for our modified rabbit ventricular model for Fig.1 in the main manuscript.
