# Supplementary material for: Two-variable nullcline analysis of ionic general equilibrium predicts calcium homeostasis in ventricular myocytes
Source: PLoS Comput Biol. 2020 Jun 5;16(6):e1007572. doi: 10.1371/journal.pcbi.1007572 (PMC7316341; doi:10.1371/journal.pcbi.1007572)
Supplement: Attachment — Submitted filename: AnswersFinal.pdf [file pcbi.1007572.s002.pdf]

Reviewer #1: The authors address the important question of how to ascertain steady state calcium conditions in a ventricular myocoyte. To this aim, they derive two coupled equations that characterise calcium equilibria. In turn, they employ this new framework to explain counter-intuitive results from SERCA therapy that is used to treat heart failure. Overall, this is an interesting study worth publishing that fits well with PLoS Computational Biology. Before making a decision regarding its publication, I would like the authors to consider the following points.

- My main comment regards the computations involved in the derivation of equations (25) and (26). As far as I understand the approach, the authors need to numerically solve the underlying large system of coupled ordinary differential equations (ODEs) to evaluate the integrals in equations such as (20). In the computation, some variables are set to their steady state values due to time scale separation, hence reducing the dimensions of the ODE system. To then determine the nullclines, the authors need to run their simulations a large number of times to determine the surfaces in Figures 3 and 4. In turn, this is used to find the fixed point as an intersection of the nullclines. If this is indeed the case, I am unclear of the advantage of the approach. If essentially, I need to run a large number of simulations (although only over one beat) to determine the fixed point, why can I not run a single simulation for longer and then determine the steady state value? In fact, Figure 4 shows that these approaches are equivalent. It would be worth explaining in more detail why computing the nullclines (which has to be redone every time parameter values are changed) is superior to running a single long simulation. For this, I also do not see how the time scale separation gives any advantage for the proposed framework as it could equally well be applied to the single long simulation.

In fact, in order to obtain the nullclines we need to run multiple one beat simulations, starting with different initial conditions. This certainly needs more computational time than running a single long simulation to compute the final steady state value. The main advantage of our formulation is not computational, but rather conceptual. Reducing the system to a state with just two variables allows the study of the structural properties of calcium homeostasis. This opens the door, for the first time, to predict all the possible outcomes from a change in parameters. Furthermore, although this case is not studied in this paper, the cross of the nullclines could give steady-state points that are not stable, which would not be accessible from simulations. Another possible scenario would be the presence of several steady states, that could result in bistability, for instance. So, we do not claim that our method is a substitute for time simulations, but rather a complement of those, that can provide a deeper understanding of the dynamics, possible steady-states and their stability, and robustness of the dynamics under changes in parameters.

As for the dimensionality reduction, we do not use it in the simulations to compute the nullclines. During one beat all variables change with time and we compute them dynamically. What we mean by dimensionality reduction is that the final state after one period changes if we change the initial values of SR and cytosolic Ca, but not if we change the initial values of other variables, that adapt fast (in a time scale faster than a period) to the state given by the chosen calcium concentrations. This is, the surfaces given by Eqs. (25)-(26) can be

parameterized by just two variables, cytosolic and SR calcium, simplifying enormously the analysis of the dynamics of the system.

- The authors balance the fluxes during one beat, which is then used to determine the steady state calcium concentrations. Another way of looking at this is to write down a map that maps the calcium concentration at the beginning of a beat to that at the end of the beat and then look for fixed points of this map. If this is indeed a sensible interpretation of the authors' work, I strongly recommend to put their results into context. Maps have a long tradition in cardiac modelling as a means to reduce the high dimensionality of cardiac models. The authors should expand on this history and also highlight how their map is different from established approaches (or how it relates to them). It is also worth mentioning that a similar idea was used in Huertas, M. A., Smith, G. D., & Gyorke, S. (2010). Ca2+ alternans in a cardiac myocyte model that uses moment equations to represent heterogeneous junctional SR Ca2+. Biophysical Journal, 99(2), 377–387. To reduce the dimensionality of calcium dynamics, a recent study proposes the use of the Master stability function on a piecewise linear version of a well-established model of calcium cycling in ventricular mycoytes: Veasy, J., Lai, Y. M., Coombes, S., & Thul, R. (2019). Complex patterns of subcellular cardiac alternans. Journal of Theoretical Biology, 478, 102–114.

The method we use is actually to compute two coupled maps, for the global SR and total calcium concentrations:

$$Q_T{}^{n+1} = Q_T{}^{n} + \Delta Q_{in}{}^{n} - \Delta Q_{out}{}^{n} = f(Q_T{}^{n}, Q_{SR}{}^{n})$$
$$Q_{SR}{}^{n+1} = Q_{SR}{}^{n} + \Delta Q_{up}{}^{n} - \Delta Q_{rel}{}^{n} = g(Q_T{}^{n}, Q_{SR}{}^{n})$$

and then analyze them to find the equilibrium conditions. The interesting (and nontrivial) part is that these maps actually describe well the dynamics, despite the model being composed of tens of thousands of stochastic elements. In similar atrial models, for instance, where spatial heterogeneity appears naturally due to centripetal propagation, we have found that these types of maps for global variables are not sufficient to describe properly the dynamics. In the case of ventricular cells, where these maps give a good description of the dynamics, we can just take arbitrary initial conditions in Q_T and Q_{SR}, *independently of the state of other variables*, and calculate their values after one beat. With this, we can uniquely compute the maps. Thus, what we do is basically the same as in Huertas et al, although they calculate the maps using a moment equation model of calcium dynamics and we use a Monte Carlo model. They calculate the SR release-load relations, once the cell has reached homeostatic equilibrium and use it to study the onset of alternans. We, by the contrary, are interested in this homeostatic equilibrium and, thus, we have to supplement the map for SR calcium concentration with another one for total cell calcium concentration. Now we explicitly write down the map equations in the Methods section and cite the works by Shiferaw et al, 2003; Huertas et al, 2010 and Qu et al, 2019, for reference on SR calcium maps.

In the paper by Veasy et al (which, in turn, is a generalization of the method developed by Li & Otani, 2003), they compute the stability of the basic periodic state by doing a linear stability analysis. The reduction of the dimensionality of the system (if we understand it properly)

comes from the fact that the CaRUs have nearest-neighbor coupling, so one can map it to a problem of independent units, and because they use a piecewise linear model. How this can be extended to continuous models with stochastic dynamics is not clear to us, although it is certainly a fascinating challenge for future work. We now make reference to these works in the discussion.

- The authors note that a particular advantage of their model is the explicit representation of buffer dynamics and not the use of the fast buffer approximation. As they point out, "The reason is that fast buffering approximation leads to a loss of mass in any type of propagation algorithm we have considered." In the limit of perfect time scale separation, i.e. infinitely rapid buffering, fast buffering is exact. While it is true that there is no ODE for the buffers, the algebraic relation following from the fast buffer approximation allows us to recover the buffered concentrations. There does not seem to be a "loss of mass" here. The question is then more if the assumptions of the fast buffering approximation are satisfied. Of course, if that is not true, then using it is misleading and might contribute to what the authors call "loss of mass". It is also unclear to me if their arguments are based entirely on a numerical implementation (as the authors make reference to the Euler scheme) or whether they believe it is a structural problem for all numerical algorithms. If it is the former, I suggest to change the integrator. Also, I think it is a misuse of terminology to call the authors' model mass conserving. The model clearly does not conserve overall mass since it neglects dynamics of the extracellular calcium concentration, which is indeed clamped. At the end of the results section, the authors state "The reason is that free calcium concentrations and total calcium concentrations can be related one-to-one, in a general equilibrium framework, using the fast-buffering approximation." Does this imply that the authors used the fast buffer approximation after all?

We, in fact, use the fast buffering approximation in the case of the buffer calsequestrin. But we do not use the reduction to an equation for free calcium, as developed in Wagner & Keitzer & 1994. We have tried to explain it more clearly in the text but we think we must explain ourselves further here. Assuming that we have only cytosolic and SR calcium, we would have the following equations:

$$\frac{\partial c_{cyt}}{\partial t} = J_{rel} - J_{up}$$

$$\frac{\partial c_{sr}}{\partial t} = \frac{v_{cyt}}{v_{sr}}(J_{up} - J_{rel}) - J_{buf}$$

$$\frac{\partial c_{buf}}{\partial t} = J_{buf}$$

Clearly, the quantity $v_{\mathrm{cyt}}c_{\mathrm{cyt}}+v_{\mathrm{sr}}(c_{\mathrm{sr}}+c_{\mathrm{buf}})$ is a conserved quantity. What we do to solve these equations in the rapid buffer approximation is to consider the system for c_{cyt} and $c_{\mathrm{sr}}^{\mathrm{tot}}=c_{\mathrm{sr}}+c_{\mathrm{buf}}$

$$\frac{\partial c_{cyt}}{\partial t} = J_{rel} - J_{up}$$

$$\frac{\partial c^{tot}{}_{sr}}{\partial t} = \frac{v_{cyt}}{v_{sr}}(J_{up} - J_{rel})$$

and then obtain the free SR calcium using the rapid buffer approximation $J_{\mathrm{buf}} \approx 0$, so

$$c_{sr}{}^{tot} = c_{sr} + c_{buf} = c_{sr} + B_T \frac{c_{sr}}{c_{sr} + K_B}$$

This formulation will conserve mass exactly in any forward integration scheme, independently on whether the fast buffer approximation is correct or not. That will only determine the relative distribution of buffered and free SR Ca.

Now, usually, the fast buffer approximation is used to obtain a dynamical equation for free SR, such that

$$\frac{\partial c_{cyt}}{\partial t} = J_{rel} - J_{up}$$

$$\frac{\partial c_{sr}}{\partial t} = \beta(c_{sr})\frac{v_{cyt}}{v_{sr}}(J_{up} - J_{rel})$$

with

$$\beta(c_{sr}) = \left(1 + \frac{B_T K_B}{(c + K_B)^2}\right)^{-1}$$

We found that mass balance is not satisfied using this formulation. The surprising fact that we have found is that this unbalance is not necessarily small. We are currently in the process of writing a report about this problem, once we understand how this effect depends on the numerical scheme used to solve the equations. Given that we do not have yet a clear picture of it, we prefer not to discuss this topic in the present manuscript. In the revised version we just state how calcium buffering is implemented pointing out the differences with other approaches and indicating how to implement calsequestrin so that the algorithm applies the rapid-buffering approximation and satisfies mass balance.

- One way of reducing the dimensionality of the model is by splitting variables into fast and slow dynamics and then express the fast variables as a function of the slow variables. Could the authors show explicitly from numerical simulations that this time scale separation holds and that they obtain the same results as with the full model, i.e. without using algebraic equations for the fast variables?

We do not actually use algebraic equations for the fast variables in the simulations. It is just that, after one period, the final state does not depend on the initial state of these fast variables, and therefore they can be neglected in our analysis. We show this now explicitly in a

figure in SI (and shown below), where we compute the end-diastolic value of SR calcium concentration after a period, starting from different initial values of the buffers TnC and CAM, and in the fraction of initial open RyRs. From the figure, it is clear that the final state is completely independent of the initial state of the buffers, and depends very slightly on the initial state of the RyRs, as long as it is close to its value at the equilibrium point. We have used this result to explain further in the SM that "The reason for this slight dependence on the state of the RyR, contrary to what happens with the buffers, is that the release depends very sensitively on the number of ready to open states of the RyR. The dynamics of both the RyR and LCC channels is fast (~100ms) compared with the pacing period, but they do not have time to equilibrate in the time between the external excitation and the initiation of release (~20ms). Thus, starting with values very far from the equilibrium value would affect the equilibrium surfaces." In any case, the fact that the predictions of the maps (or general equilibrium model) agree very well with the numerical results obtained integrating the dynamical equations is an indication that the dimensionality reduction works.

Regarding this last point we have clarified the sentence *"The reason is that free calcium concentrations...."* in the manuscript. We now clearly state that "*The reason is that end-diastolic free calcium concentrations can be related to total calcium concentrations assuming the equilibrium concentration of calcium bound to buffers. So, despite the fact that in the mathematical model we only use the fast buffering approximation for calsequestrin, the other buffers equilibrate so fast that they attain the equilibrium values by the end of each period.*"

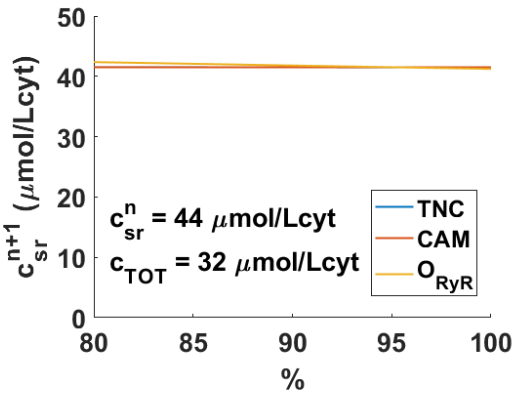

Figure: Value of SR calcium concentration after one stimulation, starting from the same initial value of c_{sr}^{n}=44µmol/Lcyt, and different initial values of buffer concentration and fraction of open RyRs. The final state is independent on the specific value at the beginning of the stimulation.

Minor comments:

- In the author summary, "2+" needs to be superscript in "Ca2+"
- l. 79: "is not a cube with". Do the authors mean that a CRU is a rectangular cuboid?
- l.157: Formally, the buffer contributions are subtracted, not added to the calcium equations.
- Equation (12) misses a bracket in the denominator.

All corrected

- l.267: Why is the unit of J_LCC mol/s and not M/s?

We have corrected the typo and now reads μmol/Lcyt/s. We thank the referee for pointing this out.

- Could the authors provide a source for the large diffusion coefficient within a z-plane? A cytosolic diffusion coefficient of 1.2 μm^2/ms (i.e. 1200 μm^2/s) appears really large (with a similar argument for the SR).

We are sorry for the mistake in the table where we mixed up a previous version with a new version. The diffusion coefficient is indeed 0.3 μm^2/ms in our simulations in the cytosol and 10 times lower in the SR. In the table we indicated 1.2 but this is diffusion *rate* between neighboring in-planes. This is, D/(dx^2)=1.2 ms{-1} . Diffusion rates across the z-planes are different than in plane. We have now included all the correct information in the new table.

Reviewer #2: This study from Conesa et al uses an exciting and original approach to predict and explain the homeostatic equilibrium in cardiac calcium handling. This approach is potentially very powerful, with the ability to provide both substantial predictive ability as well as mechanistic explanations for complex and often counter-intuitive observations

I have no major concerns with the study itself, which is in general well and clearly described, and certainly uses suitable approaches for the objectives. However, I do have some comments regarding the presentation and structuring of the paper.

Structure: I feel that different parts of this paper are placed in the wrong locations. The description of the general equilibrium approach I feel would be better suited to the methods, as this explains the framework underlying the paper – the validation of this method can then be the first part of the results. Moreover, the majority of the discussion reads more like results – presenting the applications of the validated model to explain the example of both intuitive and counter-intuitive responses to SERCA upregulation – including 2 results figures both presented within this discussion. I would suggest moving all of this to the results section (the final, summary figure should remain in the discussion).

We have restructured the paper in order to address the different points raised by the referees. We have shortened the description of the currents and buffering moving equations to the SM and placed it just below the method description of the general structure of the model. We have also included the general equilibrium approach in the method section as suggested by the referees and we start now the results with the validation process.

Furthermore, this does mean the discussion lacks some of the content which should be present. The implications for future research, in particular in context of the explanation of the SERCA gene therapy study failure, should be expanded on. Similarly, the limitations should be clearly described. There is one limitation which, while certainly not reducing the value of this study, does need to be discussed: The clamped AP; I completely understand why this was performed and that the approach would be significantly more challenging if this were not the case, but it does need to be clearly described as a limitation. In particular, changes to LCC and NCX will directly impact the AP, and changes to the CaT and its impact on LCC and NCX will also affect the AP; these AP affects may then result in further changes to the dynamics and affect the homeostatic equilibrium, as it is under different conditions. This non-linear interaction is a key component of long-term cardiac dynamics, and should therefore be explicitly discussed.

We completely agree with the referee on this point and we put the proper emphasis in the discussion. Since we have been doing preliminary tests on these effects we can address how we expect the interaction of APD and calcium homeostasis to work. We now state in the discussion:

"In this paper, we have considered a clamped AP because the APD generally adapts to changes in currents and clamping does not affect this main insight. If we were to take the proper shape of the APD for a given frequency as our clamped AP in the model, the nullclines depicted here would not be affected. However, for future work, it would be interesting to study the interplay of calcium homeostasis with changes in the AP at different frequencies in order to address

other cardiac properties such as the structure of force-frequency relations in different animal models. Since most of the adaptation of the AP is fast, we expect it to be generally slaved to the dynamics of calcium, at least, at the time scale of seconds. In this respect, a fulllinear stability analysis of the periodic transient, with an explicit calculation of the most unstable (or less stable) eigenvalues and eigenmodes \cite{li2003ion, veasy2019complex} would help to validate this point. However, there are slower times scales, associated with the long term accumulation of ions, that will become another dynamical variable, increasing the complexity of the general equilibrium problem. More specifically, one should expect the slow change of potassium and sodium concentrations to affect the LCC and NCX which in turn will affect the nullclines which will feedback into the APD and back again into the ionic balance. The general structure of our approach will hold, but a full analysis of the relevant slow variables in the full model will be needed."

Regarding the summary figure. I am happy with this as it is, but do feel it could be further improved by also having the pathway to increased transients on there. I do understand that this is more trivial, but it could really help understand the differences between these two opposing outcomes for the same input changes.

We take to heart this criticism. We have reworked figure 7 and divided it in two parts, showing better how a change in SERCA function can both increase or decrease the calcium transient depending on the homeostatic properties and nullclines structure. We have also changed the figure caption accordingly to clarify further the figure.

As a smaller comment, I think the mechanism of CICR should be introduced much earlier than its current introduction ~ ln 124. Some arguments in the intro (such as the explanation of effect on SR release) would be better supported by this basic mechanism having already been described

Point taken. We have now expanded the brief introduction of CICR in the introduction

Minor text suggestions:

We thank the referee for the revision. We have corrected all the mistakes and typos and scaled back the "perfect fit" for a "very good fit".

1. Please rephrase all "anti-intuitive" with "counterintuitive" (which is used in some cases).
2. Ln 12: maybe increasing the heart rate, rather than rhythm, would be clearer? A rhythm is regarding regularity rather than rate.
3. Ln 22: "Species dependence is not limited to the release of calcium: its reuptake into the SR …" may be a better way of phrasing this sentence.
4. Ln 33: dysregulations -> dysregulation.
5. Ln 37: "strategies as gene therapy" -> "strategies such as gene therapy". This minor error is repeated a few times: I have noted the ones I have noticed but please keep a look out for further examples.
6. Ln 64 "seem clear" -> "seems clear"

7. Ln 79. The “is not a cube” statement is a little confusing, especially given that what follows does not describe what it actually is if not a cube?
8. Ln 91: “is sensible to the calcium gradient” should be “sensitive”. This error is also made a few times within the MS, and please do not assume I have noticed every instance.
9. Ln 258: typo in equilibrium (no “b”!)
10. Ln 263: CaRU has already been defined?
11. Ln 276: “Being this the case” -> “This being the case”
12. Ln 334: Please scale back a little on the description of the fit as being “perfect”
13. ln 337: Sensible -> sensitive error
14. Ln 362: “such as macroeconomics”
15. Ln 472 “behaviour such as discordant”
16. Ln 499: “clearly and in-silico failure” needs revising.

Reviewer #3: Conesa et al have investigated calcium homeostasis in ventricular myocytes using a computational model and mathematical analysis. In general, the article was interesting, and I liked that they used economic models/approaches.

We thank the referee for the positive response and try to address below all the questions and comments. Basically we have restructured the paper clarifying the model with further references as requested. We have also clarified the notation and, more importantly, add a new paragraph to address the crucial first point and a new figure 7 plus a paragraph in order to clarify the final figure, as requested also by other referees. We have also addressed all the typos in text and figures.

I have the following questions

• Equations for JLCC and JNCX do not contain csr. Page 10, equation 25 (and Figure 4), why is it dependent on csr?

This is one of the key insights of the paper that was clearly not properly explained in he manuscript. We have added the following paragraph.

"It is rather intuitive that release and uptake depend on cytosolic and SR calcium concentration at the beginning of one beat. Less intuitive is that intake and extrusion also depends on c_{sr} *despite the fact that the L-type Calcium current and exchanger expressions do not depend instantaneously on it. The reason of this strong dependence on the SR calcium concentration at the beginning of the beat is because a larger or smaller initial calcium SR load leads to larger or smaller cytosolic calcium transients during the beat. A different calcium transient results, in general, in a different inactivation in the LCC and NCX extrusion during that beat."*

• Page 7, "We use the expression given in [26] for the properties of LCC in rabbit." However, the model in [26] has 7 states. Please provide more details on your 5-state model. Is the model validated based on experimental data?
• Similarly, the RyR model has only one open state whereas the RyR model in [26] has two open states.

We clarify now that we do not use the states inactivated by Barium in the original model published in [26] so that we reduce it to 5 states. We also use the standard RyR model developed by Stern for the RyR (Stern et al, J General Physiol, 1999) where there is one open state and we take into account the possibility of RyR termination/inactivation and includes the termination and the junctional SR dependence on RyR opening and inactivation described in Cantalapiedra et al, Chaos, 2017.

• Typical Ca_SR is 700 ~1000uM. In this study, Ca_SR is too low (<100 uM). It should be at least >500uM.

We use μmol per liter of cytosol in our graphs and analysis as used, for example, by Shannon, Ginsburg, and Bers, Biophys J, 2000. Given that the total volume of the SR is 12.5 times smaller

than the cytosol in our model, a concentration of free calcium in the SR at 40 μmol/Lcyt is roughly 500 μM at the SR level. In terms of total calcium in the SR (free and bound to buffers), this gives a value of ~100 μmol/Lcyt, that is the value obtained in Shannon et al. We consider this level to be the standard one for rabbit. A level of 20 μM/Lcyt is highly depleted SR and a value of 60 μM/Lcyt is a highly loaded SR. We clarify this point now in the caption of Figure 4.

• The organization of the paper could be changed to put the sections in a better order. For example, some materials from the "Discussion" section can be moved to the "Result" section. The "Method" section can be simplified.

We agree with the referee here and have reordered the manuscript in order to address the different points raised by the referees. In order to accommodate all criticisms in a way that we think it is better we have moved part of the method section (the sections about buffering and currents in the model) to the SM and moved the first part of the results to the methods sections since it can be understood as the development of the new methods. In the new version, both the validation and the discussion has been moved to the results section. Discussion can now fully addressed a detailed explanation of figure 7 as requested below.

• Page 2, line 12, it stated "In most animal species…" I wonder if there is any animal/mammal that the amount of blood pumped at each beat doesn't increase with beat rate?

There are some species, like mouse and rat, where the relation between contractile force and beat rate has been observed to be negative (see Antoons et al, The Journal of physiology, 2002; Georgakopoulos and Kass. The Journal of Physiology, 2001), and, certainly, some isolated cells present also even inverse relations (Ashley et al, Am J Physiol. 1999; Gattoni et al, J Physiol 594, 2016). This most probably will translate to a decreased amount of blood pumped per beat. However, since it is true that this relation is not direct (since cardiac output depends also on preload, afterload and contractile state of the heart), we have rewritten the statement to say that most animal species present important increases in blood pumped at each beat.

• Page 2, line 47, there is a typo "calcium" • Page 4, line 86, is "attached-to-buffers" same as "bound"? If yes, it would be good to be consistent and not to use different terms for the same thing.

Corrected.

Page 5, from line 134 to 152, could they cite one or two articles?

We have added two articles which are the origin of the fast buffering approximation (Wagner & Keizer, 1994; Smith, Wagner & Keizer, 1996). Besides, we have clarified the sentence *"The reason is that free calcium concentrations...."* in the manuscript. We now clearly state that " *The reason is that end-diastolic free calcium concentrations can be related to total calcium concentrations assuming the equilibrium concentration of calcium bound to buffers. So, despite the fact that in the mathematical model we only use the fast buffering approximation for*

calsequestrin, the other buffers equilibrate so fast that they attain the equilibrium values by the end of each period."

Page 9, equation 22, does inside the parenthesis (phi_i) refer to the variables of the ith CRU?

We indicated in the text that ϕ_i refer to the thousand of internal variables of the cell. The i index was not thought to be particular related with ith CRU. However, in the structure of the model the total number of variables is the sum of the variables for each CRU. Strictly one should refer to all internal variables in the cell as ϕ_j^i where j stands for a variable in the ith CRU. We use the new nomenclature in the new version of the manuscript.

• Figure 1, panel 2, it would be good to depict the subsarcolemmal space inside the cell too. Figure 1, panel 3, x-axis values are missing. Figure 2, all the panels don't have x-axis values.

We have corrected the figures adding the subsarcolemmals and nSR space and the x-axis values

• Figure 3, lower panel, in the 3D figure, what is "fmol"? Figure 5, on 3D plots, what is "fmol"?

We have clarified that fmol is femtomol, that is 10^{-15} mol in figure caption of Figure 3

Figure 4, right panel, it would good to label the curves as the f- and g-nullclines (similar to Fig S1)

We agree it is a good idea to insist on both the name and the equality behind in the figure, so we have added the name of the nullcline too.

Figure 5, for 3D plots, could they zoom in? it is hard to see what is going on there.

We have changed the distribution of the different panels so the 3D plots are clearer and larger.

Figure 6, for x-axis, could they use the notations similar to their Supplemental Material's figures (Fig S2 and S3)? In general, I found the Supplemental Material much easier to follow.

We have changed the figure as suggested.

Figure 7, there is a typo in the "SERCA Therapy" box. It is not easy to follow figure 7.

We take to heart this criticism. We have reworked figure 7 and divided it in two parts, showing better how a change in SERCA function can both increase or decrease the calcium transient depending on the homeostatic properties and nullclines structure. We have also changed the figure caption accordingly to clarify further the figure.
